# Downregulation of LncRNA GAS5 causes trastuzumab resistance in breast cancer

**DOI:** 10.18632/oncotarget.8413

**Published:** 2016-03-28

**Authors:** Wentong Li, Limin Zhai, Hui Wang, Chuanliang Liu, Jinbao Zhang, Weijuan Chen, Qun Wei

**Affiliations:** ^1^ Department of Pathology, Weifang Medical University, Weifang, Shandong Province, 261053, P.R. China; ^2^ Department of Oncology, People's Hospital of Shouguang City, Shandong Province, 262700, P.R. China; ^3^ Department of Health Care, Weifang People's Hospital, Weifang, Shandong Province, 261053, P.R. China; ^4^ Department of Molecular Genetics, Weifang Medical University, Weifang, Shandong Province, 261053, P.R. China; ^5^ Department of Pathology, People's Hospital of Shouguang City, Shandong Province, 262700, P.R. China

**Keywords:** trastuzumab, lapatinib, drug resistance, lncRNA GAS5, mTOR

## Abstract

Therapeutic resistance to trastuzumab caused by dysregulation of long noncoding RNAs (lncRNAs) is a major obstacle to clinical management of HER2-positive breast cancer. To investigate which lncRNAs contribute to trastuzumab resistance, we screened a microarray of lncRNAs involved in the malignant phenotype of trastuzumab-resistant SKBR-3/Tr cells. Expression of the lncRNA GAS5 was decreased in SKBR-3/Tr cells and in breast cancer tissue from trastuzumab-treated patients. Inhibition of GAS5 promoted SKBR-3 cell proliferation, and GAS5 knockdown partially reversed lapatinib-induced inhibition of SKBR-3/Tr cell proliferation. GAS5 suppresses cancer proliferation by acting as a molecular sponge for miR-21, leading to the de-repression of phosphatase and tensin homologs (PTEN), the endogenous target of miR-21. Moreover, mTOR activation associated with reduced GAS5 expression was required to suppress PTEN. This work identifies GAS5 as a novel prognostic marker and candidate drug target for HER2-positive breast cancer.

## INTRODUCTION

HER2 overexpression and amplification are detected in 15%–20% of breast cancers and correlate with poor prognosis and short survival [1, 2]. Anti-human HER2 antibody therapy using trastuzumab is used to treat HER2-positive, early-stage, and metastatic breast cancers but less than 35% of patients initially respond [3, 4]. The remainder are inherently resistant or acquire resistance during disease progression. Trastuzumab resistance remains an obstacle for treatment of these patients [5–7].

A recently discovered mechanism of trastuzumab resistance is the dysregulation of long noncoding RNAs (lncRNAs). Previous studies showed that lnc-ATB promotes trastuzumab resistance and invasion–metastasis cascade in breast cancer by competitively binding to miR-200c, thereby upregulating ZEB1 [8].

Novel agents targeting different signaling pathways could overcome the limitations of trastuzumab. Lapatinib is a potent and reversible inhibitor of the epidermal growth factor receptor (EGFR or ErbB1) and the ErbB2 receptor [9]. In the present study, we aim to determine the potential role of lncRNA regulation in enhanced tumor suppression activity of lapatinib in trastuzumab-resistant breast cancer cells.

## RESULTS

### GAS5 is downregulated in SKBR-3/Tr cells and trastuzumab-treated breast cancer tissues

**Figure 1:**
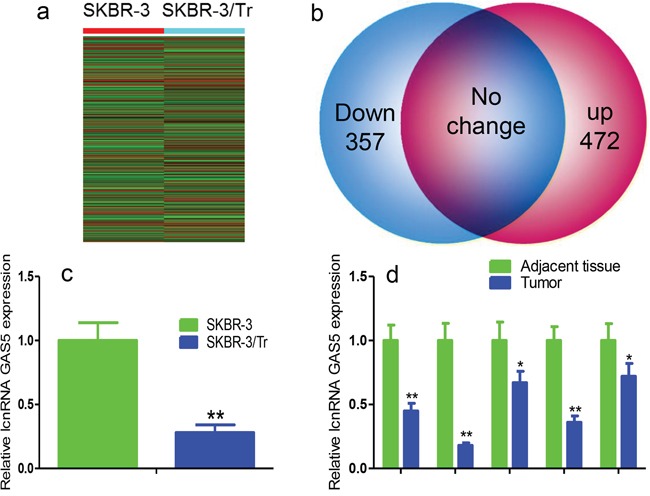
Verification of GAS5 in SKBR-3/Tr cells and trastuzumab-treated breast cancer tissues **a.** Heatmap expression profile of lncRNAs in SKBR-3 and SKBR-3/Tr cells. **b.** Diagram representations of dysregulated lncRNAs between SKBR-3/Tr and SKBR-3 cells. Note: red and blue dots represent up-regulated and down-regulated lncRNAs, respectively. **c.** Real-time PCR analysis of GAS5 expression in SKBR-3/Tr and SKBR-3 cells. **d.** GAS5 expression in tissues of breast cancer patients with or without trastuzumab treatment. Data are expressed as means ± SD. * *P* < 0.05, ** *P* < 0.01.

Microarray analysis with replicates for 10,802 lncRNAs was performed to examine differences in lncRNA expression in SKBR-3 and SKBR-3/Tr cells; the signal was normalized, averaged, and plotted. 357 lncRNAs were down-regulated and 472 were up-regulated using a twofold change as the split point (Figures 1a, 1b). The result agrees with a previously study that showed increased lnc-ATB [8]. GAS5 was among the top 15 down-regulated lncRNAs. The expression of GAS5 was confirmed by real-time PCR monitoring and normalization to 18s (Figure 1c). The results of Real-time PCR analysis showed that GAS5 expression was significantly downregulated in trastuzumab-resistant patients with breast cancer (Figure 1d).

### Downregulation of GAS5 is associated with poor prognosis of human breast cancer

**Figure 2:**
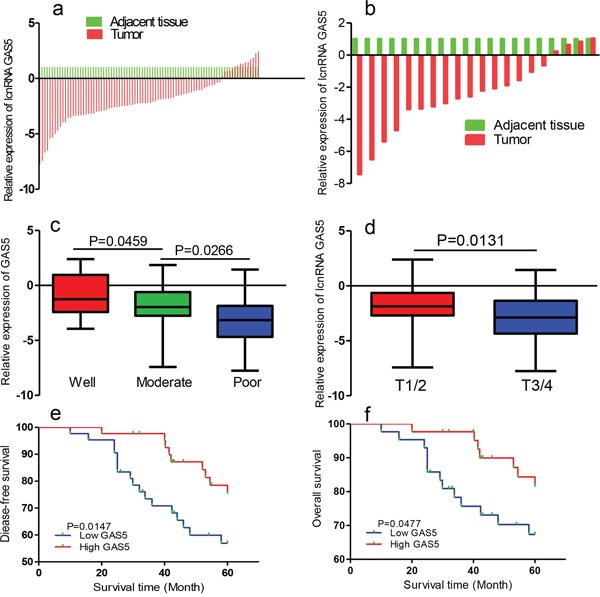
GAS5 expression is decreased in human breast cancer tissues and correlates with poor prognosis **a.** Relative expression of GAS5 in breast cancer tissues (n = 86) compared with corresponding non-tumor tissues. **b.** GAS5 levels in primary HER2-positive breast cancer tumor tissues were determined by qRT-PCR RNA. **c.** GAS5 expression was lower in patients with higher pathological stage (T3/4) than in those with lower pathological stage (T1/2). **d.** Differences in relative expression of GAS5 in breast cancer tissues among different histological classification groups. Statistical differences between samples were analyzed using the Wilcoxon signed-rank test. **e.** Kaplan-Meier analysis of disease-free survival of breast cancer patients was analyzed according to GAS5 expression levels. **f.** Kaplan-Meier analyses of correlations between the GAS5 expression level overall survival of 86 breast cancer patients.

We determined GAS5 expression in 86 pairs of breast cancer tissues and corresponding non-tumor tissues by qRT-PCR analysis. Transcript levels of GAS5 were decreased in most breast cancer tissues (Figure 2a). GAS5 expression was downregulated in all HER2-positive breast cancer specimens (n = 20) relative to pair-matched noncancerous tissues (Figure 2b).

Low expression levels of GAS5 correlated with advanced TNM stage and histological grading (*P* < 0.05, Figures 2c and 2d). Kaplan–Meier survival estimates showed that low GAS5 expression in breast cancer tissues was associated with poor disease free survival (DFS) (P = 0.015, log-rank test) and overall survival (OS) (P = 0.048, log-rank test) (Figure 2e and 2f).

### GAS5 knockdown promotes SKBR-3 cell tumorigenesis and metastatic potential *in vitro* and *in vivo*

**Figure 3:**
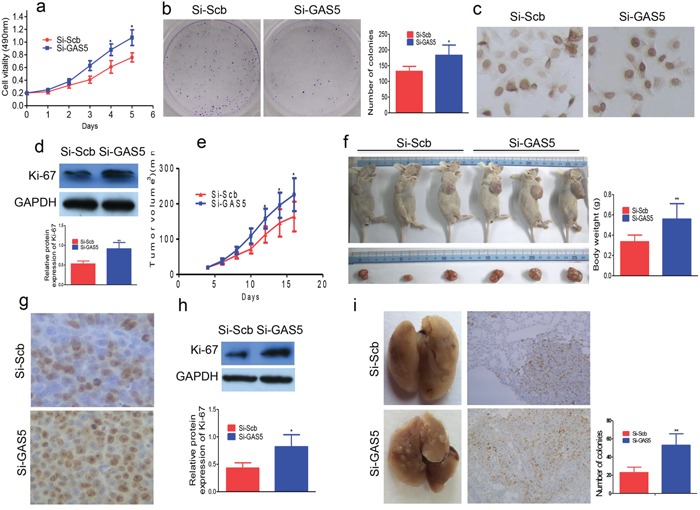
GAS5 knockdown promotes breast cancer cell proliferation **a.** GAS5 knockdown promotes the proliferation of SKBR-3 cells. **b.** The colony number of GAS5 knockdown SKBR-3 cells increased. **c.** Ki-67-positive cells were increased in GAS5 knockdown cells by immunostaining. **d.** Ki-67 expression as determined by western blot. **e.** The tumor size was monitored every two days. **f.** Mice were sacrificed and the tumors were isolated after 16 days. **g.** Transplanted tumors with Ki-67 immunostaining, Ki-67-positive cells were increased in si-GAS5. **h.** The expression of Ki-67 was also significantly increased in si-GAS5 group as determined by western blot. **i.** Left panel: Lung metastatic tumor nodules observed in lung surface of SCID mice received tail vein injection with si-GAS5 or si-Scramble SKBR-3 cells. Right panel: The expression of Ki-67 in tumor stained by IHC in lung sections. All values are presented as mean ± standard error based on at least three independent experiments. * *P* < 0.05, ** *P* < 0.01.

We transfected si-GAS5 into SKBR-3 breast cancer cells; GAS5 knockdown increased cell proliferation (Figure 3a). Colony formation assays revealed that si-GAS5 transfected SKBR-3 cells formed more colonies than control cells (Figure 3b). Immunostaining results revealed that the number of Ki-67-positive cells increased in GAS5 knockdown cells compared to si-Scramble cells (Figure 3c). Western blot analysis revealed that Ki-67 expression was higher in the si-GAS5 group (Figure 3d).

In order to confirm the effect of GAS5 *in vivo*, we subcutaneously inoculated the SKBR-3 cells transfected with si-GAS5 or si-Scramble into nude mice. Consistent with *in vitro* results, tumors in the si-GAS5 group grew more quickly up to 16 days after the injection than in the control group (Figure 3e). The average size of tumors derived from GAS5 knockdown cells increased by 66.1% (Figure 3f). Furthermore, immunostaining revealed that the number of Ki-67-positive cells were higher in tumors from GAS5 knockdown cells (Figure 3g). Western blot analysis further revealed that Ki-67 expression was higher in these tumors (Figure 3h).

To determine the role of GAS5 in tumor metastasis *in vivo*, we injected 2.5 × 10^6^ si-GAS5 stably transfected SKBR-3 cells into the tail veins of SCID mice. Five weeks after injection, the mice were euthanized. Mice that received si-GAS5 cells had more tumors on the lung surface than those administered with the control cell si-Scramble Vector (Figure 3i). Histological analysis confirmed the presence of metastatic tumors in the lungs of these mice (Figure 3i). Furthermore, there were more Ki-67-positive cells in tumors formed from GAS5 knockdown tissues (Figure 3i).

### Lapatinib alleviates trastuzumab resistance by up-regulation of GAS5

**Figure 4:**
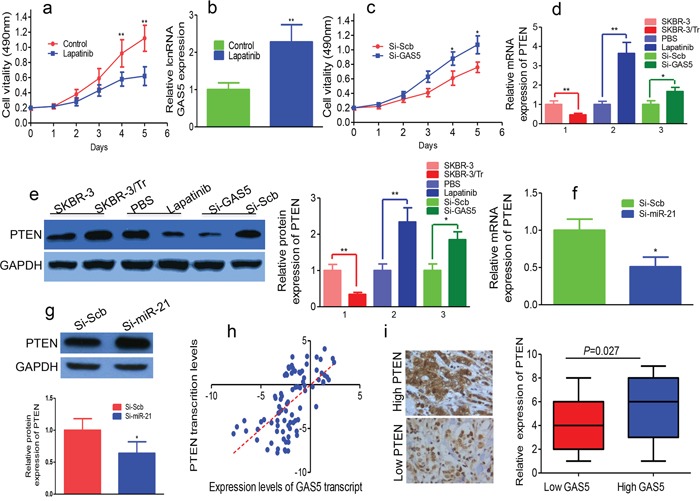
GAS5, upregulated by lapatinib, is predicted to sponge endogenous miR-21 targeting PTEN in trastuzumab resistant cells **a.** Lapatinib inhibits proliferation of SKBR-3/Tr cells. **b.** Relative expression of GAS5 in SKBR-3/Tr cells treated with lapatinib was tested by qPCR. **c.** After transfection with si-GAS5, MTT assay was performed to determine the proliferation of lapatinib-treated SKBR-3/Tr cells. **d.** PTEN mRNA was quantified by qPCR in SKBR-3/Tr cells. Lapatinib up-regulated PTEN mRNA in SKBR-3/Tr cells. GAS5 knockdown by si-GAS5 transfection reduced PTEN mRNA in lapatinib-treated SKBR-3/Tr cells. **e.** PTEN protein was downregulated in SKBR-3/Tr cells. PTEN protein was upregulated in lapatinib-treated SKBR-3/Tr cells. The levels of PTEN protein were detected by western blot in SKBR-3 cells and lapatinib treated SKBR-3/Tr cells transfected with si-GAS5. **f.** Repression of miR-21 overcame the inhibitory effects of decreasing GAS5 on the expression of PTEN mRNA. **g.** Depletion of miR-21 restored the inhibitory effects of downregulating GAS5 on cell the expression of PTEN protein by western blot. **h.** The correlation between GAS5 transcript level and PTEN mRNA level was measured in breast cancer tissues. The delta-Ct values were subjected to Pearson correlation analysis. **i.** Left panel: The high and low expression of PTEN in the breast cancer tissues. Right panel: The expression level of PTEN was higher in GAS5 high expression group than in the GAS5 low expression group. * *P* < 0.05, ** *P* < 0.01.

Lapatinib blocked the proliferation of SKBR-3/Tr cells and upregulated GAS5 (Figures 4a and 4b). MTT assays showed that knockdown of GAS5 reversed growth inhibition induced by lapatinib treatment in SKBR-3/Tr cells after the transfection (Figure 4c).

### GAS5 knockdown downregulates PTEN expression in breast cancer

PTEN is a key modulator of trastuzumab sensitivity in HER2-overexpressing breast cancer. qPCR and Western blot showed PTEN was downregulated in SKBR-3/Tr cells (Figures 4d and 4e). Lapatinib increased PTEN mRNA and protein in SKBR-3/Tr cells (Figures 4d and 4e). We transfected si-GAS5 into SKBR-3 cells and confirmed transfection by qPCR. GAS5 knockdown reduced the mRNA and protein expression levels of PTEN. Knockdown of GAS5 downregulated lapatinib-induced expression of PTEN in SKBR-3/Tr cells (Figures 4d and 4e).

### GAS5 competitively binds endogenous miR-21 targeting PTEN

We stably overexpressed si-miR-21 or si-Scramble in SKBR-3 cells. The inhibition of GAS5 decreased PTEN mRNA and protein levels, and the inhibition of GAS5 on PTEN was abolished by depletion of miR-21 (Figures 4f and 4g). The expression levels of GAS5 and PTEN mRNA in the same set of 86 breast cancer tissues were measured (Figure 4h). GAS5 transcript level correlated with PTEN mRNA level. The level of PTEN protein expression, determined by immunohistochemistry, was significantly higher in the GAS5 high-expression group compared with that in the GAS5 low-expression group (Figure 4i). Our data suggest GAS5 increases PTEN levels by competitively binding to miR-21.

### Lapatinib upregulates GAS5 in trastuzumab-resistant breast cancer through mTOR pathway

**Figure 5:**
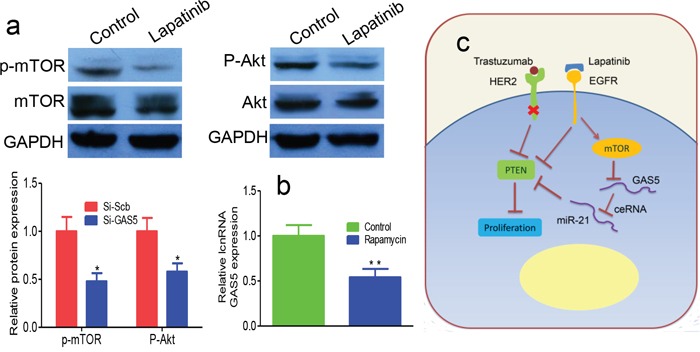
Lapatinib upregulates GAS5 through activation of mTOR pathway **a.** Lapatinib treatment increased p-Akt and p-mTOR protein expression. **b.** Rapamycin blocked lapatinib induced expression of GAS5. **c.** EGFR signaling pathway is important in the development and progression of various human tumors. EGFR strongly activates another kinase, Akt, via a lipid kinase, PI3K, leading to activation of mTOR. Activation of mTOR signaling blocks GAS5, which works as a ceRNA for miR-21 and decreases the expression of PTEN. Downregulation of PTEN favors cancer cells growth and migration. * P < 0.05, ** P < 0.01.

To analyze the signaling pathways downstream of EGFR involved in regulating GAS5, we performed Western blot assays on lapatinib-treated SKBR-3/Tr cells. p-Akt and p-mTOR protein levels were reduced in lapatinib-treated cells relative to controls (Figure 5a), while Akt and mTOR were minimally changed. This finding indicates that lapatinib may inhibit the PI3K/Akt/mTOR signaling pathway. Additionally, inhibition of mTOR by rapamycin increases the expression of GAS5 (Figure 5b). In summary, GAS5, regulated by mTOR pathway, serves as a ceRNA of miR-21 to regulate PTEN in the development of breast cancer trastuzumab resistance (Figure 5c).

## DISCUSSION

This study sought to unravel the molecular mechanisms of trastuzumab resistance. Beyond the well-known HER2 and EGFR, other pathways, such as HER3 or insulin-like growth factor receptor, activation of PI3K/Akt/mTOR, overexpression of c-MET or up-regulation of Src activity, and loss of PTEN or MUC4, are involved [12, 13].

Recent studies link lncRNAs to the development of chemoresistance. For example, a differentially expressed lncRNA ARA was validated in MCF-7/ADR and HepG2/ADR cells [14]. LncRNA-UCA1 increases cisplatin resistance during bladder cancer chemotherapy by increasing the expression of Wnt6 and thus represents a potential target to overcome bladder cancer chemoresistance [15]. lncRNA ATB is among the most upregulated lncRNAs in trastuzumab-resistant SKBR-3 cells and in the tissues of trastuzumab-resistant breast cancer patients [8].

Given their important regulatory effects on cancer progression, we infer that a relationship exists between lncRNAs and trastuzumab resistance. GAS5 was among the 15 most downregulated lncRNAs in SKBR-3/Tr cells by genome-wide microarray analysis; reduced GAS5 expression was verified by qPCR. GAS5 knockdown induced SKBR-3 cell viability and strengthened Ki-67 expression.

Knockdown of GAS5 promoted cell proliferation and tumor growth *in vivo*. GAS5 expression was markedly decreased in the tumor tissues relative to surrounding non-cancerous tissue. Low GAS5 expression in breast cancer patients correlated with histological grading and advanced TNM stage. These findings indicate that GAS5 is reduced by trastuzumab and may act as a tumor suppressive gene regulator in trastuzumab-resistant breast cancer.

To overcome the limitations of trastuzumab, new agents targeting different signaling pathways are urgently needed. The small-molecule lapatinib is classified as a dual inhibitor of ErbB1 and ErbB2, preventing downstream signaling events [9, 16]. We confirmed that lapatinib suppresses the proliferation of SKBR-3/Tr cells by upregulation of PTEN and GAS5. Lapatinib inhibition of cell growth was impaired by knockdown of GAS5 with downregulation of PTEN in this study. These findings suggest that lapatinib partially inhibits the proliferation of trastuzumab-resistant breast cancer cells through the upregulation of PTEN and GAS5. Multiple signaling pathways, such as PI3K/Akt/mTOR, mitogen-activated protein kinase, and Wnt/β-catenin, are activated by EGFR to enhance proliferation, survival, invasion, and metastasis of cancer cells [17]. We demonstrate that inhibiting mTOR signaling by rapamycin reversed the GAS5-induced upregulation of PTEN.

Given the important regulatory effects of PTEN and GAS5 on trastuzumab resistance, we infer that a relationship exists between PTEN and GAS5. Several RNA transcripts, including lncRNAs, have been identified as competing endogenous RNAs (ceRNAs), such as CCAT1 and H19 [18, 19]. Previous studies showed that GAS5 functions as a ceRNA for miR-21, and that miR-21 promoted tumor proliferation and invasion by targeting PTEN [20]. As a tumor suppressor, GAS5 enhances PTEN expression by inhibiting miR-103 expression in endometrial cancer [21]. Here, we found GAS5 functions as a molecular sponge for miR-21, increasing PTEN expression. The GAS5 transcript level was correlated with PTEN mRNA level and protein level in breast cancer tissues.

From a broader perspective, the identification of GAS5 as an important prognostic factor for breast patients warrants further investigation of its functional roles. This study may provide a strategy and facilitate the development of GAS5 directed diagnostics and therapeutics against trastuzumab-resistant breast cancer.

## MATERIALS AND METHODS

### Tissue samples and clinical data collection

Human breast cancer specimens (n = 86) were obtained from the Affiliated Hospital of Weifang Medical University. Clinicopathological data, including patient age and sex, tumor size, lymphatic invasion, lymph node metastasis, distant metastasis, pathological stages, and clinical stage, were obtained from the patient database. Normal breast tissues were collected from the same patients at a site distant from the primary tumor as control samples. This study was approved by the Research Ethics Committee of Weifang Medical University. Written informed consent was obtained from each patient.

### Cell culture, chemical, and plasmids

The HER2 overexpressing human breast cancer cell line SKBR-3 was purchased from the Chinese Type Culture Collection, Chinese Academy of Sciences (Shanghai, China) and verified by mycoplasma and cell vitality detection. Cells were cultured in RPMI 1640 medium (Invitrogen, CA, USA) and supplemented with 10% fetal bovine serum (Hyclone, MA, USA) at 37°C in a humidified air atmosphere containing 5% CO_2_. Trastuzumab (Roche, Basel, Switzerland) was dissolved in sterile water. Trastuzumab-resistant SKBR-3/Tr cells were obtained by continuous culture with 5 mg/mL trastuzumab for 6 months as previously reported [8, 10], and were cultured in RPMI 1640 medium with 250 μg/mL trastuzumab. Lapatinib was purchased from Selleck Chemicals. SKBR-3 cells or SKBR-3/Tr cells were untreated or treated with 10 μM lapatinib for 2 days. The shRNAs GAS5 (CUUGCCUGGACCAGCUUAAUU) and miR-21 (UAGCUUAUCAGACUGAUGUUGA) were cloned into the pENTER/U6 vector. The U6 promoter and interference fragment were amplified by PCR and subcloned into the SpeI site of pCDH-CMV-MCS-EF1-Puro vectors (System Biosciences, CA, USA). Lentiviruses were produced by transfecting HEK 293T with a 3-plasmid system according to the manual's instructions.

### Microarray analysis

Total RNA was extracted from the treated SKBR-3 and SKBR-3/Tr cells. lncRNAs in the RNA samples were profiled using human lncRNA microarray analysis (Kangchen Bio-tech, Shanghai, China) following the manufacturer's instructions. Quantile normalization and data processing were performed using the GeneSpring GX v11.5.1 software package (Agilent Technologies). Differentially expressed lncRNAs were identified using volcano plot filtering. The thresholds used to screen up-regulated or down-regulated lncRNAs and miRNAs were fold change ≥ 2 and *P* < 0.05.

### Immunohistochemistry and scoring system

The resected specimens were paraffin embedded and expression of PTEN and ki-67 were detected using labeled streptavidin biotin. The intensity of positive staining was determined by integrated optical density. The results were graded according to the intensity (0-negative, 1-weak, 2-moderate, and 3-strong) and percentage of positive cells [0, 1 (1–24%), 2 (25–49%), 3 (50–74%), and 4 (75–100%)], with discrepancies resolved by consensus. The grades were multiplied to determine a score.

### MTT assay and colony formation assay

Cell proliferation was tested with the tetrazolium salt 3-(4, 5-dimethylthiazol-2-yl)-2, 5-diphenyltetrazolium bromide reagent (MTT, Sigma) according to the manufacturer's instruction. Briefly, 2 × 10^3^ cells were plated in each well of the 96-well plates with 200 μL medium. Prior to analysis, 20 μL of MTT (2.5 mg/mL) was added to each well and incubated for 4 h. The medium was then removed, and the cells were solubilized in 150 μL of dimethylsulfoxide for colorimetric analysis (wavelength, 490 nm) on a microplate spectrophotometer. Growth values were calculated as follows: (OD treated cells/OD untreated cells) × 100. The experiments were performed in triplicate, and cell proliferation curves were plotted using the absorbance at each time point.

For colony formation assay, 100 cells were plated into six-well plates and incubated in RPMI 1640 with 10% FBS at 37°C. After 7 days, the cells were fixed and stained with 0.1% crystal violet. The number of colonies was counted. The experiments were performed in triplicates and repeated three times.

### qRT-PCR assay and western blot assay

For qRT-PCR, total RNA was extracted from tissues or cultured cells using TRIzol reagent (Invitrogen, CA, USA). RNA was reverse transcribed to cDNA using Reverse Transcription Kit (Invitrogen). Real-time PCR analysis was performed according to the manufacturer's instruction. The gene–specific primers used were: miR-21-F, 5′-ACACTCCAGCTGGGTAGCTTATCAGACTGA-3′, miR-21-R, 5′-TGGTGTCGTGGAGTCG-3′; GAS5-F 5′-CTTCTGGGCTCAAGTGATCCT-3′, GAS5-R 5′-TTGTGCCATGAGACTCCATCAG-3′; U6-F, 5′-CGCTTCGGCAGCACATATACTAAAATTGGAAC-3′, U6-R, 5′-GCTTCACGAATTTGCGTGTCATCCTTGC-3′; 18S-F, 5′-GTAACCCGTTGAACCCCATT-3′, 18S-R, 5′-CCATCCAATCGGTAGTAGCG-3′, PTEN-F, 5′-GTTTACCGGCAGCATCAAAT-3′; PTEN-R, 5′-CCCCCACTTTAGTGCACAGT-3′, GAPDH-F, 5′-AAGGTGAAGGTCGGAGTCAA-3′, and GAPDH-R, 5′-AATGAAGGGGTCATTGATGG-3′. Transcript quantification is shown as mean ± standard error from three independent experiments performed in triplicate.

For Western blot analysis, cell protein lysates were separated by 12% SDS-polyacrylamide gel electrophoresis (SDS-PAGE), transferred to 0.22 μm PVDF membranes (Sigma, MO, USA), and incubated with specific antibodies. GAPDH antibody was used as control. Autoradiograms were quantified by densitometry. Anti-Ki67 and anti-PTEN antibodies were purchased from Santa Cruz Biotechnology, Inc. (CA. USA). All experiments were conducted three times, and the median and mean values were calculated.

### Animal studies

Female athymic BALB/c nude mice were housed under pathogen-free conditions and manipulated according to standard protocols. SKBR-3 cells stably transfected with si-GAS5 or si-Scramble were harvested from six-well cell culture plates and resuspended at a concentration of 1 × 10^8^ cells/mL. 100 μL of the suspended cells were subcutaneously injected into a single side of the posterior flank of each mouse. Tumor growth was examined every 3 days, and tumor volumes were calculated using the equation V = 0.5 × D × d^2^ (where V, volume; D, longitudinal diameter; d, latitudinal diameter) [11]. 15 days post-injection, mice were euthanized, and subcutaneous growth of each tumor was examined.

Female SCID mice (4 weeks old) were obtained from the Animal Center of the Chinese Academy of Science (Shanghai, China) and housed in sterile laminar flow cabinets. At the age of 6 weeks, the mice were injected with 2.5 × 10^6^ cells of SKBR-3-si-GAS5 or SKBR-3-si-Scramble through the tail vein. After 5 weeks, the SCID mice were euthanized. The lungs were removed and photographed. Obvious lung metastatic tumors on the surface were examined and quantified. Lung tissues were placed in 4% paraformaldehyde and paraffin-embedded for H&E staining and immunohistopathological examination.
